# Polymorphisms of inflammatory and metalloproteinase genes, *Helicobacter pylori* infection and the risk of oesophageal adenocarcinoma

**DOI:** 10.1038/sj.bjc.6604234

**Published:** 2008-02-05

**Authors:** M Früh, W Zhou, R Zhai, L Su, R S Heist, J C Wain, N S Nishioka, T J Lynch, F A Shepherd, D C Christiani, G Liu

**Affiliations:** 1 Medical Oncology, Department of Medicine, Princess Margaret Hospital, University of Toronto, 610 University Avenue, Toronto, Ontario, Canada M5G 2M9; 2 Department of Environmental Health, Harvard School of Public Health, 665 Huntington Avenue, Boston, MA 02115, USA; 3 Department of Medicine, Massachusetts General Hospital Cancer Center, Harvard Medical School, 665 Huntington Avenue, Boston, MA 02115, USA; 4 Department of Surgery, Massachusetts General Hospital, 10 Blossom Street, Boston, MA 02114, USA; 5 Endoscopy Unit, Department of Medicine, Massachusetts General Hospital, 10 Blossom Street, Boston, MA 02114, USA; 6 Pulmonary Unit, Department of Medicine, Massachusetts General Hospital, 10 Blossom Street, Boston, MA 02114, USA; 7 Applied Molecular Oncology, Ontario Cancer Institute, 610 University Avenue, Toronto, Ontario, Canada M5G 2M9

**Keywords:** polymorphism, metalloproteinases, oesophageal cancer, *Helicobacter pylori*

## Abstract

*Helicobacter pylori* (HP) infection appears protective against oesophageal adenocarcinoma (EA) risk. Matrix metalloproteinases (MMPs) are released in the presence of HP infection. In *MMP2* wild-type individuals, HP was significantly protective of EA risk (adjusted odds ratio: 0.29; 95% confidence interval=0.1–0.7). Matrix metalloproteinases may modulate the EA–HP relationship.

The increasing incidence of oesophageal adenocarcinoma (EA) (Younes *et al*, 2002) may be explained partly by the widespread nature of chronic gastro-oesophageal reflux disease (GERD) and Barrett's oesophagus (Kim *et al*, 1997; Shaheen and Ransohoff, 2002). Epidemiologic studies suggest that *Helicobacter pylori* (HP) infection protects against EA (Chow *et al*, 1998; de Martel *et al*, 2005) and GERD (Raghunath *et al*, 2003). A postulated mechanism for the protective effect involves acid reflux reduction with HP-mediated chronic atrophic gastritis (Sozzi *et al*, 1998).

A number of factors influence the chronic atrophic gastritis severity. Myeloperoxidase (MPO) released from inflammatory cells and manganese superoxide dismutase 2 (SOD2) enhance inflammation and tissue damage, thereby increasing the severity of chronic atrophic gastritis (Suzuki *et al*, 1995; Smoot *et al*, 2000). In addition, the expression of matrix metalloproteinases (MMPs), responsible for the degradation of extracellular matrix components, is increased during HP infection, thereby enhancing chronic atrophic gastritis (Bergin *et al*, 2004).

The *Val* allele of the *SOD2* −*Ala16Val* polymorphism results in decreased SOD2 enzyme transport into mitochondria (Shimoda-Matsubayashi *et al*, 1996). Likewise, the *A* allele of *MPO −463 G/A* is associated with lower MPO enzyme levels (Piedrafita *et al*, 1996). The *2G* allele of *MMP1 −1607 1G/2G* polymorphism is associated with higher MMP1 expression levels (Rutter *et al*, 1998). The *T* allele of *MMP2 −1306C/T* disrupts an Sp1 regulatory element leading to lower promoter activity and decreasing MMP2 expression (Price *et al*, 2001). In contrast, the *MMP3 1171 −6A/5A* polymorphism causes transcriptional elevation and modulates expression of *MMP3* (Ye *et al*, 1996). *MMP12 −A82G A* allele is associated with a higher *MMP12* promoter activity (Jormsjo *et al*, 2000).

We hypothesise that genetic variants associated with greater inflammatory gene and higher MMP expression are associated with a greater extent of chronic atrophic gastritis and greater protection against the risk of EA.

## MATERIALS AND METHODS

### Study population

Local institutional review boards approved the study. All cases gave written consent, were >18 years old and were diagnosed within the last 6 months. All had histologically confirmed EA that was deemed endoscopically (or at the time of resection) to have a tumour centre located at or above the midpoint of the gastro-oesophageal junction, with at least two-thirds of the tumour bulk located in the oesophagus (Liu *et al*, 2007). The serum samples of 100 cases that were collected and processed in a uniform manner were analysed. They had similar age, gender and disease distribution as the 83 recruited cases that were not analysed due to serum collection problems (*P*>0.10 for each comparison). A total of 101 age- and gender frequency-matched healthy controls were composed of lifetime cancer-free, GERD-free, non-blood-related family members (usually spouses) and friends of other cancer/surgical patients. For all participants, a standardised interviewer-administered questionnaire collected information on age, gender, race, body mass index, smoking status/history and HP infection status. Body mass index in the third decade of life was used as a surrogate of healthy adult weight decades prior to the development of EA. Over 90% of the participants were born in the United States.

### Genotyping and HP determination

DNA was extracted using the Puregene DNA Isolation Kit (Gentra Systems, Minneapolis, MN, USA). The *MMP1 −1607 1G/2G*, *MMP2 −1306C/T*, *MMP3 −6A/5A* and *MMP12 −82A/G* polymorphisms were genotyped by 5′-nuclease assay (TaqMan) using the ABI Prism 7900HT Sequence Detection System (Applied Biosystems, Foster City, CA, USA); conditions and primers are available upon request. *SOD2 Ala16Val* and *MPO −463 G/A* genotyping was performed as described previously (Liu *et al*, 2004).

Serum was processed within 1 h of collection and stored at −70°C, with no freeze–thaw cycles prior to analyses, and was run in mixed blinded batches. *Helicobacter pylori* infection status and CagA status were evaluated using Helicoblot 2.1 (Genelabs Diagnostics®, Singapore City, Singapore and Redwood City, CA, USA).

For quality control determinations of laboratory techniques, positive and negative controls and blinded duplicate samples were run. Alternative genotyping approaches (different primers, endonucleases, techniques, conditions) to verify technical reliability and accuracy were used as required. A second scientist checked all laboratory interpretation independently, and a blinded third scientist arbitrated discrepancies.

### Statistical analysis

Univariate and bivariate explorations of the data were performed. Case and control demographic data were compared using Fisher's exact and Student's *t*-tests, where appropriate. If homozygous variant genotype frequencies were very low, heterozygous and homozygous variant genotypes were combined. Hardy–Weinberg equilibrium was tested using the *χ*^2^ test. Using standardised kit criteria, we defined four different categories of HP infection status based on the antibodies detected with the Helicoblot 2.1 kit: ever infection, current infection, infection with CagA or infection with VacA strains (Monteiro *et al*, 2001; Hoang *et al*, 2006).

Analyses of associations between the different genetic polymorphisms, HP infection status and EA risk were based on logistic regression models (SAS, version 9.1, SAS Institute Inc., Cary, NC, USA). Where appropriate, crude and adjusted odds ratios (AOR) and 95% confidence intervals (CI) for the risk of EA were calculated. For adjusted analyses, we included adult body mass index, smoking status, age and gender. To test for a gene–HP interaction, an interaction term was added to the logistic regression model (likelihood ratio tests). The SAS macro HAPPY was used for haplotype determination and *D*′ calculation.

## RESULTS

Baseline demographics are shown in Table 1. The recruitment rate was >85% for cases and controls. Cases had the following stages: I (*n*=9), IIA (*n*=18), IIB (*n*=17), III (*n*=22), IVA (*n*=5) and IVB (*n*=29). The proportions of cases and controls treated for HP infection were 16 and 12%, respectively (*P*>0.30). There were no overall associations between each polymorphism and the EA risk. The overall AOR between HP infection and EA was 0.71 (95% CI=0.4–1.0). Duplicate genotyping was performed for at least 30% of subjects for each polymorphism with 100% concordance. Serologic analysis had 99% concordance of 100% duplicates.

The strongest and most consistent association was found between the *MMP2 −1306* polymorphism and HP infection: in 58 cases/56 controls carrying the *MMP2 −1306C/C* wild-type genotype, having HP infection at any time in their life was strongly protective against EA (AOR 0.29, 95% CI=0.1–0.7). In contrast, in 35 cases/43 controls carrying *MMP2 C/T* or *T/T* (associated with lower promoter activity), this protective effect was lost (AOR 1.76; 95% CI=0.06–5.2; for ever infection with HP). When we specifically analysed different definitions of HP status such as current, VacA+ or CagA+ infections for their HP–EA risk association, the protective effect of HP remained significant in the wild-type genotype of *MMP2 −1306C/*C (AOR ranged from 0.16 to 0.35) and abrogated in patients carrying any variant allele. The corresponding interaction model and interaction term were statistically significant (*P*<0.001). When using several other definitions of HP infection status, the *MMP2–*HP interaction terms were similarly significant: *MMP2* and CagA+ infection (interaction term, *P*=0.03), and *MMP2* and current HP infection (interaction term, *P*=0.005). Thus, both stratified analysis and interaction models point to an *MMP2–*HP relationship.

When evaluating other MMP polymorphisms, ever infection with HP was also associated with a significantly decreased EA risk in the subset of patients who carried the wild-type genotypes, *MMP3 −1171 6A/6A* (AOR 0.04, 95% CI=0.002–0.9, *P*=0.04; 27 cases/29 controls) and *MMP12 −82 A/A* (AOR 0.44, 95% CI=0.2–0.8, *P*=0.02; 75 cases/79 controls), but not for their corresponding variant genotypes. The *MMP1 −1607*, *MMP3 −6A/5A* and *MMP12 −82A/G* polymorphisms are in linkage disequilibrium (*D*′=0.5 for *MMP1-MMP3*; *D*′=0.8 for both *MMP1-MMP12* and *MMP3-MMP12*), but none of the evaluated polymorphisms are linked with the *MMP2 −1306 C/T* polymorphism (*D*′<0.15 for all comparisons). Haplotype analyses of the *MMP1-MMP3-MMP12* polymorphisms resulted in weaker associations when compared with the analyses of individual polymorphisms (data not shown).

No significant protective effects of HP infection on the EA risk were found with any genotype subsets of *MPO −463 G/A* or with *SOD2 Ala16Val*.

## DISCUSSION

To our knowledge, the question of gene–HP interaction in EA risk has not been addressed. The protective effects of HP infection on the risk of developing EA have been postulated, based on a number of epidemiologic studies (Chow *et al*, 1998; de Martel *et al*, 2005). This protection is believed to result mainly from decreased acid reflux following HP-mediated chronic atrophic gastritis (Sozzi *et al*, 1998). As only one in 200 individuals with Barrett's oesophagus develop EA annually, gene–environment factors may hold the key to understanding EA risk. Yet few studies have examined gene–environment interactions in oesophageal cancer; most were conducted in Asia, evaluating squamous cell carcinoma of the oesophagus, genetic polymorphisms and either smoking or alcohol consumption (Lee *et al*, 2001; Yu *et al*, 2004; Lin *et al*, 2006; Hiyama *et al*, 2007). We compared a set of EA cases with frequency-matched controls.

The most significant finding of our study was the increased protection against the risk of developing EA by HP infection in individuals carrying the wild-type *MMP2 −1306 C/C* genotype, whereas no protective effect could be detected among the variant genotypes. MMP2 is part of the MMP family, a group of enzymes responsible for the degradation of extracellular matrix components. MMP2 levels were increased in the presence of HP infection and contributed to tissue damage in HP-associated gastritis (Bergin *et al*, 2004). The wild-type genotype is associated with higher promoter activity and *MMP2* expression (Price *et al*, 2001). Higher MMP2 expression may lead to increased severity of chronic atrophic gastritis and subsequently less acid reflux and decreased EA risk (Sozzi *et al*, 1998; Raghunath *et al*, 2003; Bergin *et al*, 2004). Our results are therefore consistent with this mechanism. This protective effect against EA risk was maintained even when different definitions of the HP infection status were evaluated. Our examination of polymorphisms of two other *MMPs* led to similar results.

Our results should be interpreted cautiously, given the modest sample size and multiple comparisons, although even a Bonferroni correction would still leave the primary gene–HP interaction and stratified wild-type models statistically significant. In addition, the serologic evaluation of prior HP infection over time is imperfect, and may be affected by antibiotic treatment. Nonetheless, because not just one but several *MMP* polymorphisms were associated independently with gene–HP interactions, this is a novel finding that warrants further exploration.

In conclusion, we were able to demonstrate for the first time a modulation of the protective effect of HP on EA risk by several polymorphisms in the MMP pathway. These intriguing results will need confirmation in a larger prospective setting, particularly one that also explores the relationships between Barrett's oesophagus, GERD and EA prognosis.

## Figures and Tables

**Table 1:** Demographic information by case–control status

**Characteristics**	**Controls (*n*=101)**	**Cases (*n*=100)**	***P*-value**
*Gender* a			
Female	13 (13%)	12 (12%)	0.9
Male	88 (87%)	88 (88%)	
			
*Age (years)* b	63±8	64±8	0.4
			
*Race*			
Caucasian	101 (100%)	100 (100%)	NA
			
*Smoking status* a			
Non-smokers	27 (27%)	16 (16%)	0.07
Ex-smoker	54 (53%)	67 (67%)	
Current smoker	20 (20%)	17 (17%)	
			
Packyears^c^	37±31	42±36	0.5
			
*BMI (kg m* ^*−2*^ *)*			
BMI at diagnosis^b^	28±5	26±5	0.004
BMI in healthy adult^d^	22±4	23±4	0.4
			
*HP infection status* a			
HP ever infected	43 (43%)	36 (36%)	0.4
HP never infected	58 (57%)	64 (64%)	
			
*CagA strains*			
Positive	30 (30%)	29 (29%)	0.8
Negative	71 (70%)	71 (71%)	
			
*VacA strains*			
Positive	20 (20%)	15 (15%)	0.4
Negative	81 (80%)	85 (85%)	

BMI=body mass index; HP=*Helicobacter pylori*; NA=not applicable.

a Cases and controls were compared using Fisher's exact tests.

b Data are reported as mean (s.d.), and compared using Student's *t*-tests.

c In ever smokers only.

d BMI in the third decade of life (twenties).
